# Control of Cholesterol Metabolism Using a Systems Approach

**DOI:** 10.3390/biology11030430

**Published:** 2022-03-11

**Authors:** Dorota Formanowicz, Marcin Radom, Agnieszka Rybarczyk, Krzysztof Tanaś, Piotr Formanowicz

**Affiliations:** 1 Department of Medical Chemistry and Laboratory Medicine, Poznan University of Medical Sciences, 61-701 Poznan, Poland; doforman@ump.edu.pl; 2 Institute of Computing Science, Poznan University of Technology, 60-965 Poznan, Poland; marcin.radom@cs.put.poznan.pl (M.R.); agnieszka.rybarczyk@cs.put.poznan.pl (A.R.); krzysztof.tanas@cs.put.poznan.pl (K.T.); 3 Institute of Bioorganic Chemistry, Polish Academy of Sciences, 61-704 Poznan, Poland; 4 Faculty of Electrical Engineering, Gdynia Maritime University, 81-225 Gdynia, Poland

**Keywords:** cholesterol metabolism, atherosclerosis, mathematical modeling, systems biology, Petri nets, t-invariants

## Abstract

**Simple Summary:**

Cholesterol is the main sterol in mammals that is essential for healthy cell functionining. It plays a key role in metabolic regulation and signaling, it is a precursor molecule of bile acids, oxysterols, and all steroid hormones. It also contributes to the structural makeup of the membranes. Its homeostasis is tightly controlled since it can harm the body if it is allowed to reach abnormal blood concentrations. One of the diseases associated with elevated cholesterol levels being the major cause of morbidities and mortalities worldwide, is atherosclerosis. In this study, we have developed a model of the cholesterol metabolism taking into account local inflammation and oxidative stress. The aim was to investigate the impact of the interplay of those processes and cholesterol metabolism disturbances on the atherosclerosis development and progression. We have also analyzed the effect of combining different classes of drugs targeting selected components of cholesterol metabolism.

**Abstract:**

Cholesterol is an essential component of mammalian cells and is involved in many fundamental physiological processes; hence, its homeostasis in the body is tightly controlled, and any disturbance has serious consequences. Disruption of the cellular metabolism of cholesterol, accompanied by inflammation and oxidative stress, promotes the formation of atherosclerotic plaques and, consequently, is one of the leading causes of death in the Western world. Therefore, new drugs to regulate disturbed cholesterol metabolism are used and developed, which help to control cholesterol homeostasis but still do not entirely cure atherosclerosis. In this study, a Petri net-based model of human cholesterol metabolism affected by a local inflammation and oxidative stress, has been created and analyzed. The use of knockout of selected pathways allowed us to observe and study the effect of various combinations of commonly used drugs on atherosclerosis. The analysis results led to the conclusion that combination therapy, targeting multiple pathways, may be a fundamental concept in the development of more effective strategies for the treatment and prevention of atherosclerosis.

## 1. Introduction

### 1.1. Research Context

A complex network of interacting processes maintains cholesterol metabolism. Hence, these biological mechanisms should be seen and analyzed as a complex system using appropriate methods. Such methods have been developed for years in the area of systems sciences, mainly in the context of technical systems. However, recently, complex biological phenomena have been studied from the point of view of systems science, resulting in the emergence of a rapidly developing branch of science called systems biology. The main motivation for investigating biological objects as complex systems is the growing belief that many crucial properties of these objects (e.g., organs, tissues, cells, processes, etc.) not only follow from properties of their elementary building blocks, but also, or rather most of all, from structures of dens networks—of interactions among them [1].

The first (and necessary) step in analyzing a complex system involves the construction of a formal model describing it. Such a model can be expressed in the language of some branches of mathematics. Traditionally differential equations are used for this purpose, but recently, models based on graphs or networks have been frequently used. They can describe relations between elements of the analyzed system in a natural way. Among various types of models of this kind, the ones based on Petri nets seem to be especially promising. The structures of these nets are very well suited for describing structures of biological systems. Moreover, Petri nets have intuitive graphical representations, which are very useful in building the models and their interpretations. On the other hand, there are many mathematical methods and software tools used in the analysis of properties of such nets. These properties correspond to some biological features of the modeled system [1,2].

Models expressed in the language of the Petri nets theory are qualitative. It could be seen as a drawback, but it is rather an advantage in the context of biological systems. It follows from the fact that the structure of a biological system often determines many of its crucial properties. Hence, understanding this structure is essential for understanding the nature of the analyzed biological phenomena. Moreover, there are many extensions of Petri nets that allow taking into account various types of quantitative data. Thus, it is possible to start the description and analysis of a biological system with a qualitative model. When some quantitative data are available, extending this model using an appropriate extension of Petri nets and including these data are possible. What is important, the structure of the Petri net usually remains unchanged [2,3].

Although the pathways of cholesterol metabolism are widely known, and it is known that atherosclerosis is an interaction of inflammatory, oxidative, and lipid disorders, treatment of atherosclerosis remains a challenge. Therefore, we decided to use a systems approach to understand this problem better and understand the relationships within the cholesterol metabolism in the human body. Treating cholesterol metabolism as a system of interrelated interactions in which blocking one pathway may change other pathways has allowed us to observe which paths are more important and need to be blocked to stop atherosclerosis progression.

To our knowledge, there is no research focused on human cholesterol metabolism, including mechanisms underlying atherosclerosis, which would simultaneously consider so many signaling pathways and fundamental processes involved in this complex phenomena, within a single project. One of the reasons for this is that methods based on the Petri net theory allow to create molecular interaction networks with no quantitative parameters, contrary to ODE-based or PDE-based methods, where the exact values of some parameters corresponding to the quantitative properties of the system are mandatory. Since reliable reaction data are most often not accessible in the literature, the construction of ODE/PDE models is usually a difficult task, especially in a case of complex biological systems. Therefore, they are often limited to small networks where continuous kinetic changes of certain aspects of a given process are modeled [4,5,6]. There exist many mathematical models that describe the plaque growth and its stability in the arteries [7,8,9,10,11]. All of them indicate the essential role of major cholesterol metabolism elements, such as low density lipoprotein (LDL) particles, in determining whether plaque will grow or shrink, which is consistent with the results of our analysis. Other models focus on blood dynamics in rigid walls or consider compliant vessels in the framework of fluid-structure interaction [12,13,14,15].

At the same time, there is growing literature in the mathematical modeling of cholesterol metabolism formulated and studied, mainly in terms of linear and nonlinear ordinary differential equations, reviewed in [16]. Unfortunately, most of those models failed to correctly predict the response to statin therapy [16]. Another model was considered a general exogenous and endogenous cholesterol pathway within a hepatocyte, developed using the theory of nonlinear deterministic ordinary differential equations, parametrized with dimensional and non-dimensional parameter values obtained from [17], and solved using the MATLAB stiff differential equation solver ODE15s. The simple model proposed by the authors was tested against basic intervention strategies and the sensitivity analysis showed that the model in general, quantitatively reproduced the known biology of lipoprotein uptake and cholesterol regulation [18]. Many other mathematical models of selected aspects of cholesterol biosynthesis have been formulated, which differ in the range of levels of details and complexity [19,20,21,22,23].

Nevertheless, a whole-body mathematical model of atherosclerosis integrating fundamental processes underlying this disease, together with complex processes responsible for cholesterol biosynthesis and metabolism, is still missing.

### 1.2. Biological Background

Cholesterol is an essential component of cell barrier formation and signal transduction. It is involved in many fundamental physiological processes: (1) it is an essential lipid constituent of cell membranes; (2) the precursor of steroid hormones and bile acids; (3) the intermediates of cholesterol biosynthesis are required to make vitamin D and for post-translational modification of membrane proteins; (4) it is involved in atherosclerosis promotion. Therefore, its metabolism must be strictly controlled. Every cell in vertebrates has machinery for cholesterol synthesis and metabolism. Under physiological conditions, the body’s cholesterol level is relatively constant due to many regulatory mechanisms that maintain the balance between the de novo synthesized cholesterol pool, the bile cholesterol pool and the absorbed cholesterol in the intestine pool [24].

#### 1.2.1. Cholesterol de Novo Synthesis

##### Endogenous Cholesterol Synthesis

Cholesterol homeostasis is reached via tight regulation between synthesis, dietary absorption, bile salt utilization, and excretion. These pathways are regulated by three feedback mechanisms: (1) the auto-negative regulation of hepatic bile salt synthesis; (2) the positive regulation of intestinal bile salts for cholesterol absorption; and (3) excretion. Endogenous cholesterol is synthesized in enzymatic reactions, occurring mainly in the liver, intestines, and skin. The cycle of these reactions is initiated by acetyl coenzyme A (acetyl-CoA) formed by oxidative decarboxylation of pyruvic acid or beta-oxidation of fatty acids. Acetyl-CoA reacts in the cytosol with the acetoacetyl-CoA molecule, in a reaction catalyzed by 3-hydroxy-3-methylglutaryl coenzyme A synthase (HMG-CoA synthase), to form 3-HMG-CoA. Acetoacetyl-CoA is formed by condensing two acetyl-CoA molecules catalyzed by thiolase (acetyl coenzyme A acetyltransferases (ACAT)). Next, 3-HMG-CoA is reduced to mevalonate by 3-hydroxy-3-methylglutaryl coenzyme A reductase (HMG-CoA reductase, HMGR). Drugs, such as statins—which lower levels of cholesterol—stop the production of mevalonate by inhibiting HMGR. Next, the mevalonate is converted to 3-isopentenyl pyrophosphate via adenosine triphosphate (ATP)-dependent phosphorylation reactions. This multi-step transition from acetyl-CoA to 3-isopentenyl pyrophosphate is one of the three most essential cholesterol synthesis pathways [25].

In the second important cholesterol pathway, from six molecules of 3-isopentyl pyrophosphate, squalene is formed. The next important step is the cyclization of squalene (via squalene epoxide) to lanosterol and cholesterol. These reactions are catalyzed by various enzymes, such as reductase and HMG-CoA synthase, farnesyl acid phosphate synthase, and squalene synthase. The cholesterol formed in this way is the so-called pool of free cholesterol, i.e., unesterified with long-chain fatty acids [26,27].

De novo cholesterol synthesis occurs mainly if there is limited pool of lipoproteins available to the cells. In turn, the excess of cholesterol in the cells’ cytoplasm contributes to the inhibition of intracellular cholesterol synthesis, primarily by inhibiting the activity of HMGR, which prevents the excessive accumulation of cellular cholesterol. If cells do not use cholesterol, it is stored as cholesterol esters in their cytoplasms.

##### HMGR Activity Regulation

The regulation of HMGR activity is controlled by four distinct mechanisms: (1) feedback inhibition, (2) expression control, (3) enzyme degradation, and (4) HMGR covalent modification occurring due to phosphorylation and dephosphorylation processes. The first three mechanisms (1–2) are triggered by cholesterol. HMGR is most active in its unmodified form, while phosphorylation lowers its activity. HMGR is phosphorylated by 5${}^{\prime }$AMP-activated protein kinase (AMPK). In turn, AMPK itself is also activated by phosphorylation. This process requires at least two enzymes: liver kinase B1 (LKB1) and calmodulin-dependent protein kinase-β (CaMKKβ). CaMKKβ induces AMPK phosphorylation in response to an increase in intracellular calcium. Moreover, HMGR activity is further regulated by cAMP. The increased cAMP activates protein kinase A (PKA), phosphorylates the phosphoprotein phosphatase inhibitor-1 (PPI-1) and increases HMGR activity. PPI-1 can inhibit the activity of many different phosphatases, including protein phosphatase 2C (PP2C) and protein phosphatase 2A (PP2A), which removes phosphates from AMPK and HMGR. Certain hormones, such as glucagon and adrenaline, negatively affect cholesterol synthesis by increasing PPI-1 activity. In turn, insulin activates HMGR by removing phosphate [28]. HMGR activity is regulated by sterol response element (SRE), a DNA consensus sequence [29].

#### 1.2.2. The Interactions between Enterohepatic Cholesterol and Bile Metabolism

Cholesterol homeostasis is reached via tight regulation between synthesis, dietary absorption, bile salt utilization, and excretion. These pathways are regulated by three feedback mechanisms: (1) the auto-negative regulation of hepatic bile salt synthesis, (2) the positive regulation of intestinal bile salts for cholesterol absorption, and (3) excretion [24,30]. Intestinal epithelial cell cholesterol absorption is a important source of cholesterol in the human body. Hence, diet plays an essential role in the prevention and treatment of cholesterol disturbances [31]. However, mainly triglycerides (TAG) are delivered to the body this way, while dietary cholesterol supplies only 30% of the small intestine’s cholesterol pool. Another 1/2 to 3/4 of this pool is cholesterol, secreted by the liver with bile salts. About 95% of the intestine’s bile acid pool is absorbed and transported in the blood back to the liver. The remainder of the cholesterol (about 20% of the intestinal pool) comes from exfoliating epithelial cells [32].

Due to the presence of bile acids and phospholipids, free cholesterol in the intestinal lumen is emulsified, accumulated in micelles, and this form penetrates through the brush border into the intestinal epithelial cells. The flow of cholesterol from the intestinal lumen to the enterocytes is controlled by the membrane protein Niemann–Pick C1-Like 1 (NPC1L1), which is highly expressed in the small intestine [33] and liver cells [34]. NPC1L1 promotes cholesterol uptake via endocytosis. Next, from endocytic vesicles, cholesterol is transferred to the endoplasmic reticulum by the the microsomal triglyceride transfer protein (MTTP). In turn, apical sodium-dependent bile acid transporter (ASBT) [35] plays a pivotal role in the transport of cholesterol present in bile. The free cholesterol from enterocytes is then “pumped” back into the small intestine lumen via transporters—ATP-binding cassette sub-family G member 5/member 8 (ABCG5/G8). It can also be esterified by acyl-CoA: cholesterol acyltransferase (ACAT) [36]. The formed cholesteryl esters (CEs), together with the TAG, through the microsomal triglyceride transfer protein (MTTP), are incorporated into chylomicrons (CMs) [37]. The newly formed CMs, in addition to a considerable amount of TAG, CE, and phospholipids, contain several apolipoproteins (APO), including APOB-48, APOA-I and APOA-IV, which stabilize the newly emerging CMs. High lipid meals enhance the intestinal expression of APOA-IV [38].

TAG from the diet must first be hydrolyzed by lipoprotein lipase (LPL) in the intestinal lumen to pass from the lumen of the small intestine to the enterocyte cells and then be incorporated into the CM molecule. In this way, monoacylglycerols (MAG) and free fatty acids (FFA), which freely pass into the intestinal epithelial cells, are formed in the small intestine lumen. Here, in the reaction catalyzed by microsomal acyl-CoA acyltransferase–diacylglycerol acyltransferase (DGAT), they are re-synthesized into TAG [39]. The newly formed CMs are released from enterocytes by exocytosis and reach the blood through the lymphatic circulation. Here, by exchange from high-density lipoproteins (HDL), they receive APOC-II and APOE, in exchange for APOA-IV and become mature CM. The enrichment of CM with APOC-II, a cofactor for the LPL capillary endothelial cells, initiates a lipolytic cascade in which the CM TAGs are hydrolyzed multiple times. FFA formed in this way are taken up mainly by adipose tissue cells and striated muscle cells. LPL synthesis by adipose tissue cells and skeletal muscle cells is regulated by cell metabolism and satiety/hunger states. On the surface of these cells, there are heparan sulfate proteoglycans (HSPGs), capable of capturing and degrading the released LPL.

In turn, the LPL, which has avoided degradation, binds to the VLDL receptor [40] located on the capillary endothelial cell’s basal surface and is transported across the endothelium (transendothelial transport) to their lumen. Interactions between APOC-II, APOA-V and the LPL and the capillary endothelium that initiate the hydrolysis of the TAG contained in CM have been presented in detail in [41]. This hydrolysis reduces the size of the CM molecule. Reduced CM, containing mainly APOB-48, cholesterol esters, small amounts of TAG, APOE, and LPL, are called chylomicron remnants (CMR). The CMRs are small enough to squeeze between the endothelial cells lining the liver capillaries and enter the Disse space. CMRs in the liver capillaries can enter the liver parenchymal cells differently. The first, receptor-mediated endocytosis (RME), occurs through the hepatic LDL receptor-related protein 1 (LRP-1), ligands of which include LPL and APOE-rich lipoproteins [42] or via hepatic low-density lipoprotein (LDL) receptor (LDL-R) which recognize APOE on the CMR surface [43]. Moreover, CMR endocytosis is also possible due to interactions between heparin sulfate proteoglycans (HSPGs), mainly syndecans and glypicans of the basement membrane of hepatic parenchymal cells, which act as membrane receptors, and LPL and APOE present on the CMR surface. In turn, the second possibility of CMR transfer to liver parenchymal cells occurs in two stages. First, CMRs are sequestered by extracellular HSPGs such as collagen XVIII, agrin and perlecan, and thus enter the Disse space. Sequestration occurs through the reaction of LPL and APOE with the extracellular HSPG. The hepatic lipase (HL), located in the Disse space, creates an additional junction between the extracellular HSPG and CMR. Subsequently, CMR may undergo RME via LDL-R, LRP-1, or HSPG of hepatic parenchymal cells’ basement membrane [41].

Increased content of lipids and carbohydrates in the diet, exceeding the requirements of the human body cells, leads to their transformation into TAG in the liver. The endogenous TAG are taken from the plasma, with the participation of MTTP, along with the liver synthesized “de novo” cholesterol, are packed into very-low-density lipoproteins (VLDL); released into the blood and in this way transported to various organs, mainly skeletal muscle cells and adipose tissue cells. Here, they are stored or used for energy production. An essential process of lipid catabolism is the hydrolysis of TAG. The released here fatty acids are transformed into diacylglycerols, ceramides, and long-chain acyl-CoAs, playing many critical regulatory functions. The enzyme responsible for this process is hormone-sensitive lipase (HSL), activated by phosphorylation mainly with PKA [44]. The key to initiate and maintain VLDL synthesis in the hepatic endoplasmic reticulum is the availability of TAG and APOB-100 synthesized in the liver [45]. In addition to TAG and APOB-100, VLDL contains cholesterol esters, free cholesterol, APOE, APOC-I, APOC-II, APOC-III. Newly formed VLDLs, similar to newly formed CMs, acquire APOC and APOE mainly from exchanges between the HDL-2 molecule [46]. In this way, newly formed VLDLs are transformed into mature VLDLs.

The current state of knowledge on the VLDL synthesis and its regulation has been presented in detail in [45,47]. Enhanced VLDL synthesis in the liver leads to increased activity of the cholesterol ester transfer protein (CETP), responsible for transporting cholesterol esters from HDL and LDL to VLDL and the transfer of TAG and phospholipids from VLDL to HDL and LDL. As a result of the hydrolysis of TAG contained in VLDL and mentioned exchanges between lipoproteins, smaller and smaller molecules enriched in cholesteryl esters, the so-called residual VLDLs, intermediate-density lipoproteins (IDLs) are formed. The lower TAG content compared to VLDL and the absence of APOC-II characterize IDL. Hydrolysis in VLDL is similar to that in CM under the influence of LPL. It should be noted that, under the influence of the exchanges among VLDL, LDL, and HDL particles, due to TAG’s increased content, become more susceptible to the HL action and show a reduced ability to uptake cholesterol from the tissues. Moreover, they also have a shorter half-life, leading to lowering their blood levels.

Similarly, LDL particles with increased TAG content become more susceptible to HL. Their intense hydrolysis leads to the formation of small dense low-density lipoproteins (sdLDL), distinguished by a lower affinity for the LDL receptor and increased susceptibility to oxidation. The fate of IDL molecules can be two-fold. Half of them are taken up in the liver directly by RME via LDL-R or LRP-1. Under HL’s influence, the remaining IDL molecules lose other TAGs, APOC-III, and APOE, and transform into LDL lipoproteins. Cholesterol in LDL accounts for about 2/3 of all cholesterol in circulating lipoproteins. LDL lipoproteins are taken up via RME by cells with LDL-R on their surface. In these cells, the collected LDL lipoproteins are stored and only later used or are immediately converted into steroid hormones or bile acids. Part of the LDL particles is captured by the LRP-1 receptor or scavenger receptors (SR), mainly class A (SR-A), found in varying amounts on the surface of macrophages and Kupffer cells [48]. SREBP upregulates transcription of LDL cholesterol in the cells that do not synthesize cholesterol themselves.

The molecular regulation of HDL is complex as evidenced by their relations with many proteins, bioactive lipids and non-coding RNAs [49]. HDL particles, synthesized both in the liver and in the intestine, are responsible for the reverse transport of cholesterol in the human body. The HDL-2 molecule is much larger compared to the HDL-3 and contains more cholesteryl esters. In circulation, HDL particles initially appear in the form of discoidal precursors synthesized by the liver and the intestine. Newly formed HDL (nascent HDL) particles can readily take up free cholesterol from other cholesterol- and TAG-rich lipoproteins. Free cholesterol uptake is achieved by APOA-I, APOA-IV, and the ATP-binding cassette transporter (ABC-A1) named cholesterol efflux regulatory protein (CERP) [50,51]. ABC-A1 mediates the transport of cholesterol, phospholipids, and other lipophilic molecules across cell membranes into the cells’ interior, from where they are then removed as HDL particles. APOAI is the major HDL apolipoprotein and is responsible for activating the enzyme lecithin: cholesterol acyltransferase (LCAT), which "esterifies" the free cholesterol taken up and, thus, facilitates its transport [52]. Nascent HDL, under the influence of LCAT, are transformed into large mature migrating HDL-3 particles. The latter in the blood are enriched with APO and TAG, which are released during intravascular lipolysis. In this way, HDL-3 is converted to HDL-2 [53]. The distribution of lipids in HDL is opposite to their distribution in the previously described VLDL and LDL. HDLs contain only a tiny amount of TAG and many cholesteryl esters and free cholesterol, which increases their ability to take cholesterol from other lipoproteins [54].

The intake of free cholesterol increases the size of the HDL particle. The reverse transport of cholesterol can follow three pathways [55]. The first involves the uptake of HDL-2 particles with hepatic LDL-R. The second concerns the uptake of cholesteryl esters from HDL-2 by scavenger receptors B type 1 (SR-B1) located on the surface of many cells, including liver and adrenal cells [56,57]. The phospholipid transfer protein (PLTP) and HL play an essential role in this process [58]. PLTP can convert the HDL molecule into both large and small HDL particles. This process takes place in two stages [59]. In contrast, HL’s mechanism of action is based on the hydrolysis of TAG and phospholipids contained in HDL, resulting in a population of smaller HDL particles [60]. HDL2, devoid of excess cholesterol esters, can return to circulation as HDL-3 and serves as an acceptor of cellular cholesterol. The third possibility is the transfer of CE from HDL to CMR and VLDL via the CETP participation [61].

In addition to participating in the re-transport of free cholesterol from peripheral cells to the liver, HDL lipoproteins also show other anti-atherosclerotic effects, i.e., antioxidant [62], and anti-inflammatory [63,64]. HDL are complex particles that undergo dynamic changes through interactions with various enzymes and tissues throughout their life cycle. This makes it more complicated to fully understand their functions than initially thought [65].

**Figure 1:**
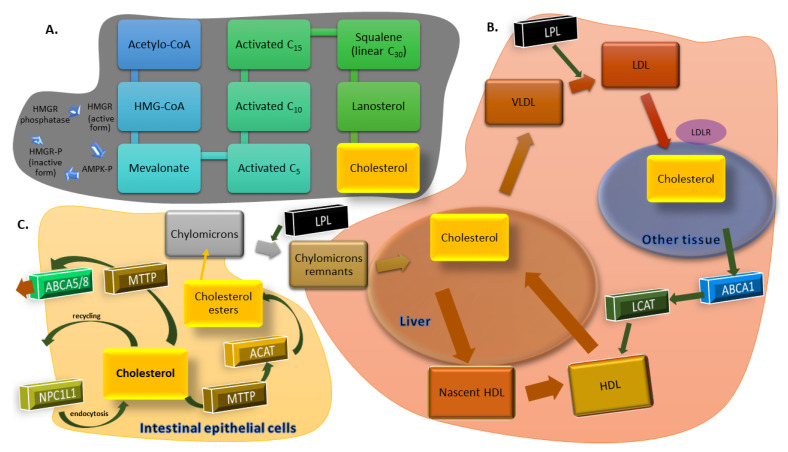
(**A**) Cholesterol synthesis. (**B**) Transport of cholesterol between the liver and peripheral tissues. (**C**) Uptake of cholesterol by intestinal epithelial cell.

A diagram showing the key processes of cholesterol metabolism has been shown in Figure 1.

However, it should be emphasized that today researchers are far from recognizing HDL-C only as “good cholesterol”. Thanks to modern techniques, they have started to notice the diversity of the HDL molecules. Although previous evidence from epidemiological studies indicated that HDL-C levels are inversely related to cardiovascular risk and that they can be used to predict risk, it has been shown that interventions to raise HDL-C levels do not provide better protection against cardiovascular diseases (see [66] for a review).

#### 1.2.3. The Role of Cholesterol in Aterosclerotic Plaque Formation

Since the cholesterol balance is closely related to oxidative stress and local inflammation [67,68], these two disorders have been also included in our model, thus reflecting what is happening at the base of the atherosclerotic plaque. Atherosclerosis plaque formation begins with a subendothelial accumulation of cholesterol-carrying LDL that stimulates innate and adaptive immune responses. LDL, especially oxidized LDL (ox-LDL), exhibit damage-related molecular pattern properties and induce activation of endothelial cells, thereby inducing an inflammatory response. Endothelial activation, through the development of local pro-inflammatory cytokines, and under influence of the oxidative stress, triggers the expression of leukocyte adhesion molecules on endothelial cells and consequently monocytes adhesion to the endothelium, see [69]. This is followed by the transmigration of monocytes via endothelial cells to the intima, where they differentiate into macrophages. In the next step, T cells bind to macrophages in the intima. Finally, the macrophages containing the modified lipoproteins (modified via oxidative stress) become lipid-rich foam cells. This local inflammatory process stimulates the migration and replication of vascular smooth muscle cells that accumulate in the plaque to form a fibroproliferative lesion. The macrophages present in the plaque show abnormal lipid metabolism with a reduction in cholesterol efflux, which leads to the accumulation of apoptotic bodies and necrotic debris forming a necrotic core in the plaque [70].

**Figure 2:**
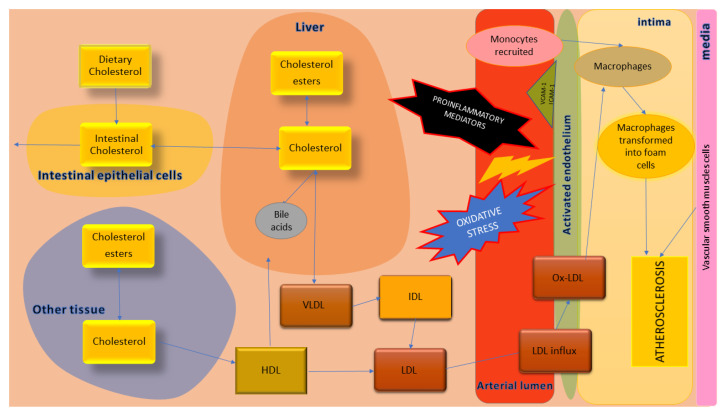
The key processes of atherosclerotic plaque formation with particular emphasis of cholesterol metabolism.

A diagram showing the key processes of atherosclerotic plaque formation has been shown in Figure 2.

In the proposed Petri net-based model of cholesterol metabolism, local inflammation and oxidative stress have been taken into account to reflect their impact on cholesterol metabolism pathways in the human body. Three main processes, i.e., cholesterol metabolism disturbances, inflammation and oxidative stress, interplay and ultimately impact atherosclerotic plaque formation.

This study aimed to check, based on the behavior of the developed model, how—by blocking individual elements in the network—one can influence cholesterol metabolism in order to achieve a state in which atherosclerosis does not arise.

## 2. Methods

### 2.1. Petri Nets

Petri nets are mathematical objects whose properties make them very well suited for modeling complex systems composed of many concurrent processes. For decades they have been used for modeling and analysis of technical systems but it appeared that they are also a very useful tool for investigating the biological ones.

Petri nets have structures of weighted directed bipartite graphs, which means that they are composed of vertices and arcs. The set of vertices is divided into two disjoint subsets in such a way that arcs can connect only vertices belonging to different subsets. Moreover, each arc is labeled by a positive integer number called a weight. Vertices, being elements of one of these subsets, are called "places", and they usually correspond to elementary passive components of the modeled systems. Vertices belonging to the other subset are transitions and they correspond to active components of the system, which usually are some elementary processes. In the context of biological systems, places may correspond, e.g., to chemical compounds or molecular complexes while transitions may be counterparts of chemical reactions or some interactions among such complexes. In such a case, places may describe substrates and products of reactions modeled by transitions. Moreover, arcs indicate causal relations between passive and active components of the system [2,71].

One of the most important and useful properties of Petri nets is dynamics. Obviously, the bipartite graph is a completely static object, so the dynamic is connected with another type of components of Petri nets, called tokens. Usually, they correspond to amounts of passive system components represented by places. Tokens reside in places and flow from one place to another through transitions, which corresponds to a flow of substances, information, etc. in the modeled system. A distribution of tokens over a set of places, called marking, corresponds to a state of the modeled system.

More formally, a Petri net is 5-tuple $Q=(P,T,F,W,M_{0})$, where $P=\{p_{1},p_{2},\cdots ,p_{n}\}$ is a set of places, $T=\{t_{1},t_{2},\cdots ,t_{m}\}$ is a set of transitions, F⊆(P×T)∪(T×P) is a set of arcs, $W:F\to Z^{+}$ is a weight function and $M_{0}$ is an initial marking [2,3,72].

The flow of tokens is governed by simple the so-called firing rule. According to it a transition is active if in all its pre-places, i.e., those ones which directly precede this transition (place $p_{i}$ precede transition $t_{j}$ if arc $\left(p_{i},t_{j}\right)$ exists), the number of tokens is equal to at least the weight of an arc connecting the places with the transition. More formally, if $m(p_{i})$ denotes the number of tokens residing in place $p_{i}$ and $w(p_{i},t_{j})$ is a weight of arc $\left(p_{i},t_{j}\right)$, then in order to activate transition $t_{j}$ it is necessary that $\underset{i\in \{1,2,\cdots n\}}{\forall }\underset{\left(p_{i},t_{j}\right)}{\exists }m\left(p_{i}\right)\geq w\left(p_{i},t_{j}\right)$, where $w(p_{i},t_{j})=0$ if arc $\left(p_{i},t_{j}\right)$ does not exists. An active transition can be fired what means that tokens can flow from its pre-places to its post-places, i.e., those ones that directly succeed this transition (more formally place $p_{k}$ is a post-place of transition $t_{j}$ if arc $\left(t_{j},p_{k}\right)$ exists). The number of flowing tokens is equal to a weight of a given arc. From this, it follows that the number of tokens flowing into a transition may be different than the number of tokens flowing out of this transition.

In a graphical representation of Petri nets transitions are depicted as rectangles or bars, places as circles, arcs as arrows connecting places with transitions or transitions with places, tokens as dots or positive integer numbers located in places and weights as positive integer numbers associated with arcs (if a weight is equal to 1 it is usually not shown in the graphical representation of the net) [2,3,72].

#### 2.1.1. t-Invariants

The above-mentioned graphical representation is very intuitive and useful, especially at the stage of model development and simulation, but it is not very well suited for formal analysis of the properties of the net. For this purpose, another representation, called an incidence matrix, is usually used. Matrix *A* of this type is composed of *n* rows (corresponding to places) and *m* columns (corresponding to transitions).

Entry $a_{ij}$ of matrix *A* is equal to a difference between the number of tokens in place $p_{i}$ before and after firing transition $t_{j}$, i.e., $a_{ij}=w\left(t_{j},p_{i}\right)-w\left(p_{i},t_{j}\right)$, where if a given arc does not exists in the net, its corresponding weight is equal to 0, as previously indicated.

In the context of biological systems, what is especially important is the analysis of a model based on t-invariants (transition invariants). An invariant of this type is vector $x\in Z^{m}$ being a solution of equation

A·x=0

To every t-invariant *x* there corresponds set $s\left(x\right)=\left\{t_{j}:x_{j}>0,j=1,2,\cdots ,m\right\}$ of transitions called a support of *x*. If every transition $t_{j}\in s\left(x\right)$ is fired $x_{j}$ times then the marking of the net is not changed. It means that supports of t-invariants correspond to subprocesses, which do not change a state of the modeled system [2,71].

An analysis of interactions of such subprocesses may lead to discovering of some unknown properties of the modeled biological system. These interactions may be looked for by searching for similarities among t-invariants. Supports of similar t-invariants can have non-empty intersections, whose elements (i.e., some transitions) correspond to elementary processes which are common for subprocesses modeled by similar invariants. These subprocesses may interact with each other via the common elementary processes. Such interactions may be a source of some previously not known properties of the system, what makes the t-invariants based analysis so important. A net is covered by t-invariants if every transition belongs to at least one support of some t-invariant. In this way, every reaction represented by a transition has some influence on the model of the biological system [71].

#### 2.1.2. MCT Sets

On the basis of t-invariants, transitions can be grouped into maximal common transition sets (MCT sets) [73,74]. They are disjoint subsets of transitions which therefore divide the net structure into some subnets. A set of this type contains transitions which belong to supports of exactly the same t-invariants. More formally, $\underset{m\in M}{\forall }\underset{t_{i},t_{j}\in T}{\forall }\left(t_{i}\in m\wedge t_{j}\in m\right)\Leftrightarrow \underset{x\in X}{\forall }\left[\left(t_{i}\in s\left(x\right)\wedge t_{j}\in s\left(x\right)\right)\vee \left(t_{i}\notin s\left(x\right)\wedge t_{j}\notin s\left(x\right)\right)\right]$, where M is a collection of all MCT sets, while *X* is a set of all t-invariants. Each MCT set corresponds to some functional module of the modeled system whose biological meaning can be determined [2,71,75]. Every transition belongs to exactly one MCT set but some of these sets can contain only one transition. Such single-element sets are called trivial MCT sets and they will not be considered in the presented analysis, since those sets do not contain any interesting information. It should be noted that an MCT set not necessarily has to induce a connected subnet, i.e., a subnet, where for any transition there exists a path containing exactly one place, connecting it with some other transition from the same set. If an MCT set contains a transition for which such a path does not exist, such a set induces a non-connected subnet. One example of such a set is present in the analyzed model and it will be mentioned in the Results and Discussion section.

#### 2.1.3. Knockout Analysis

A knockout analysis based on t-invariants for the presented model has been done using Holmes software [76]. Since all transitions are grouped into either trivial or non-trivial MCT sets, knockout analysis for the latter has also been included. Details concerning this approach on an example of Duchenne muscular dystrophy have been described in [77]. In general, in such an approach, some set of transitions has been marked as being ’knocked-out’ (disabled) and t-invariants have been recalculated for such a modified Petri net (i.e., the net without the knocked-out transitions). The resulting set of t-invariants has been smaller and the modified net has not always been covered by them. From such a knockout analysis additional knowledge about the model behavior could be acquired. For example, one could knock out some specific MCT sets or some other subset of transitions, recalculate the t-invariants and then check how many other reactions (either MCT sets or single transitions) have been affected, i.e., are no longer covered by the new, recalculated t-invariants set. On the opposite, some transition $t_{x}$ is not considered to be affected by (manually disabled, knocked-out) transition $t_{y}$ in such a knockout analysis, if $t_{x}$ belongs to a support of at least one t-invariant that does not contain $t_{y}$ in its support (therefore such a t-invariant will not be affected by disabling $t_{y}$).

Another type of knockout analysis performed in our studies has been based on the net simulation. In such an approach for a given number of steps a dynamics of the net has been studied, i.e., transition firings and token accumulations in places have been observed. This type of knockout has given additional information about the importance of some reactions, not only by telling how many other elementary processes have stopped, but also by providing data about changes in firing probability for all transitions in response to the knockout of some transition (which stopped producing tokens). Since the proposed net is not a stochastic one, a more basic simulation has been assumed. In such a simulation every active transition has 50% chance of firing. In a single simulation step, multiple active transitions can fire, under the assumption that their token requirements for firing can be fulfilled in a given net state (i.e., marking). In this type of knockout analysis, two sets have been compared. One called reference set contains data about the net behavior where nothing is disabled. For a given number of steps in a single simulation phase, the data have been gathered concerning the firing of transitions and tokens accumulation. Such simulations have been repeated a given number of times with the same starting state (i.e., when nothing is disabled). In such a way, data about transition firings and tokens in places could be averaged, thus creating the reference set.

The second so-called knockout set contains similar types of data, but it has been created with different starting conditions, i.e., some transitions have been manually disabled so they have not produced nor consumed tokens. The same number of steps and the same number of simulations have been repeated as in the case of creating the reference set, so the data could again be averaged in the knockout set. These sets have been compared and the differences in the net behavior have been analyzed, e.g., about the changes in transition average firing or about the changes of total accumulated tokens in some specific places.

## 3. Results and Discussion

### 3.1. The Model Presentation and the Results of Its Formal Analysis

**Figure 3:**
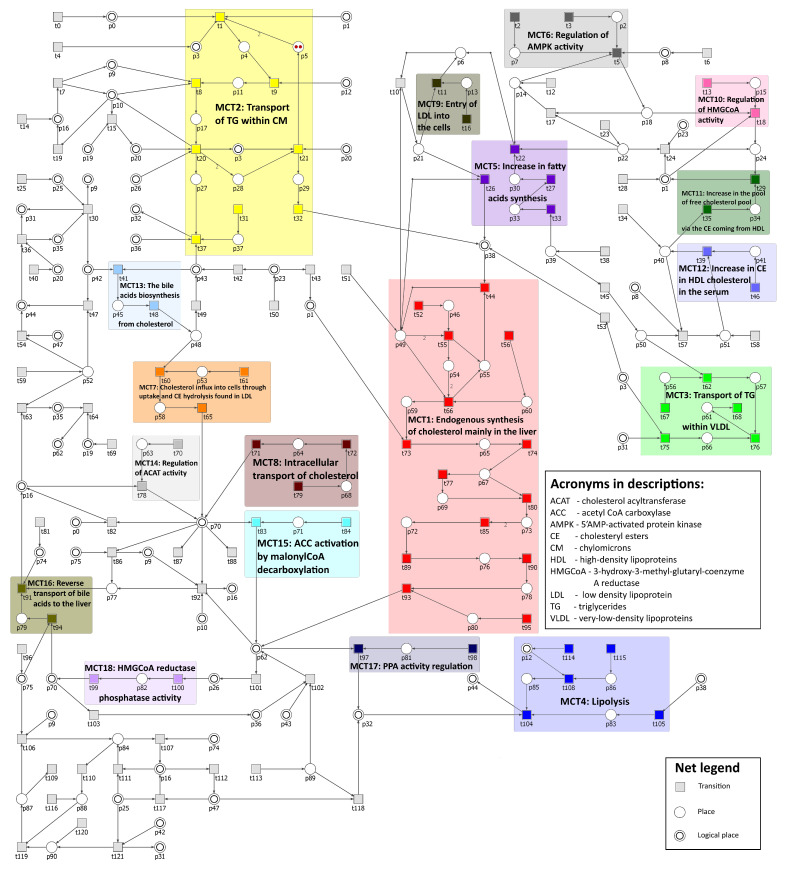
The Petri net based model of the cholesterol metabolism with MCT sets marked with different transition colors.

The Petri net-based model of the cholesterol metabolism presented in Figure 3 and available in Supplementary Materials consists of 122 transitions and 91 places. They are listed in Table 1 and Table 2 respectively.

In Figure 3, transitions and places have been aligned in "row order" of increased numbers, meaning that, for example, transition $t_{0}$ and place $p_{0}$ are in the upper left corner of the figure, then in the same row on the right, there are transitions $t_{1}$, $t_{2}$, and $t_{3}$ and places $p_{1}$ and $p_{2}$, and so on, for easier distinction within the picture. Some places in Figure 3 are depicted as two concentric circles and they are called logical places. Two or more such places with the same number are in fact different graphical representations of the same place, e.g., place $p_{0}$ have two different locations—one in the upper left corner of the figure, while its second symbol is directly connected with transition $t_{82}$.

The net is covered by t-invariants and their total number is 3871. On the basis of t-invariants, MCT sets have been calculated. A total number of non-trivial MCT sets is 18. Only one MCT set ($m_{5}=\left\{t_{22},t_{26},t_{27},t_{33}\right\}$) induces a disconnected subnet, all other sets induce subnets that are connected, as explained in the Methods section. Transition $t_{26}$ is connected with the rest of $m_{5}$ set by a path, which leads from $t_{22}$ through $p_{6}$, $t_{10}$, and $p_{21}$ (which has an arc directed into $t_{26}$). However, $t_{10}$ does not belong to $m_{5}$; therefore, this MCT set induces a disconnected subnet. All MCT sets are described in Table 3.

### 3.2. The Knockout Analysis Based on t-Invariants

**Scenario** **1.** *The analysis of the importance of each functional biological unit (MCT set) and some selected transitions of the studied model.*

At the beginning, every MCT set (including trivial ones, i.e., single transitions) has been knocked-out to answer a question of an importance of each functional biological unit of our model. This can be answered by giving the number of transitions affected by such a knockout. Affected transitions are the ones that are present in supports of affected t-invariants (i.e., t-invariants having the knocked-out transitions in their supports) and only in them. For example, a transition $t_{x}$ is not considered to be affected by $t_{y}$ if it belongs to a support of at least one t-invariant, which does not contain $t_{y}$ in its support. The same is true for non-trivial MCT sets. The results are given in Table 4 (only single transitions or non-trivial MCT sets with an impact higher than 2% of affected transitions has been shown).

From the obtained results, one can clearly see that only few transitions and MCT sets can affect a significant part of the net when being knocked-out. Transitions $t_{38}$, $t_{4}$, and $t_{45}$ seem to have the most influence, i.e., when knocked-out, each of them can disable almost one-third of the net transitions. Another observation concerns the MCT sets. From their definition, there comes a "fact" in which their transitions always appear together in t-invariants. Therefore, knocking out any transition from an MCT set will affect all of the other ones from the given set. Therefore, in Table 4, two values are given for the MCT sets. The first one tells about the percentage of all disabled transitions including the one within such a set. The second value in parenthesis tells how many transitions outside of the disabled MCT set are also affected. One can see that a knockout of sets $m_{1},m_{2}$, $m_{5}$, $m_{7}$, or $m_{8}$ only affects transitions belonging to these sets. In summary, one can clearly observe that only a few transitions (out of a total of 122) have a significant impact on the net when knocked-out. Transitions $t_{38}$, $t_{4}$, and $t_{45}$ are the most important ones. MCT set $m_{3}$ can also disable a significant part of the net if any of its transitions is knocked-out (27.9%), but it should be noted that most of the disabled transitions are the ones belonging to this particular MCT set (i.e., 23.8% of all disabled transitions belong to $m_{3}$).

**Scenario** **2.** *The influence of the major cholesterol metabolism particles on the atherosclerosis development and progression.*

Next, a knockout experiment has been performed on a specific parts of the model, representing major cholesterol metabolism elements: low-density lipoproteins (LDLs), very low-density lipoproteins (VLDLs), intermediate-density lipoproteins (IDLs), high-density lipoproteins (HDLs), and chylomicron molecules (CMs). Places representing them are listed in Table 5.

The next performed knockout experiment involved disabling the sources of specific cholesterol molecules in the model. This type of knockout requires removing the transitions that are responsible for the production of the given molecules and recalculating the t-invariants set. The results are given in Table 6, where the numbers in the last two columns are, respectively, the numbers of remaining t-invariants (from the total of 3871 ones), and the numbers of the remaining t-invariants that contain transition $t_{109}$ (atherosclerosis) in their support (434 out of 3871).

**Figure 4:**
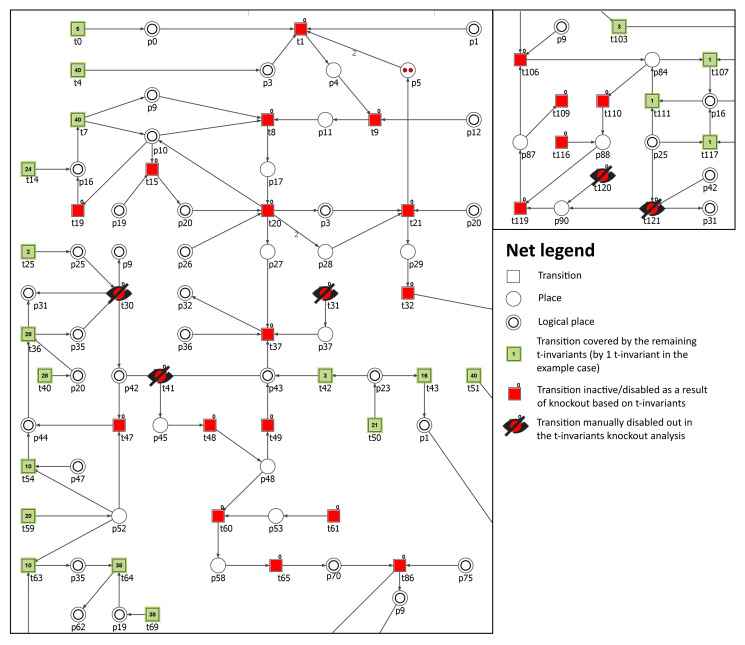
Graphical representation of the t-invariant based knockout impact of the following transitions: $t_{30}$, $t_{31}$, $t_{41}$, $t_{120}$, and $t_{121}$ on atherosclerosis ($t_{109}$) progression. The knocked-out transitions are denoted with crossed-out black circles. Transitions belonging to a support of any t-invariant are represented as filled green rectangles. The number inside the rectangle corresponds to the number of supports of t-invariants to which a given transition belongs. Transitions that do not belong to a support of any t-invariant are represented as red rectangles. The results were obtained using Holmes software [76].

As one can see from the results in Table 6, the impact depends on the cholesterol molecule whose production has been disabled. For example, to turn off the production of LDL in the model, the following transitions has been knocked-out: $t_{30}$ (conversion into LDL), $t_{31}$ (LDLR synthesis), $t_{41}$ (binding LDL and LDLR), $t_{120}$ (oxidation) and $t_{121}$ (degradation). As a result, the number of t-invariants dropped to 78. All t-invariants with atherosclerosis transition ($t_{109}$) have been disabled. The net is no longer fully covered by the t-invariants and the areas, which are not covered as a result consist of 22 transitions, among them are the ones in MCT sets $m_{2}$, $m_{7}$, and $m_{13}$. It should be noted that the net without any knockout consists of 3871 t-invariants and among them there are 434 contributing to atherosclerosis progression. One can see from Table 6 that disabling any cholesterol molecule has a major impact on $t_{109}$ (the percentage of remaining t-invariants corresponding to the processes that contribute to the atherosclerosis development and progression in the system is very low). In summary, we can say that LDL, as expected, is the most important factor in cholesterol progression—knockout of transitions contributing to its production completely turns off all of the processes involved in the atherosclerosis progression. On the other hand, knocking out the chylomicron molecule (CM) will disable most of the t-invariants involved in atherosclerosis, but at the same time, 1136 t-invariants remain as active processes. In Figure 4, an impact of such a knockout is presented.

**Scenario** **3.** *The influence of the HMG-CoA reductase related phenomena on the atherosclerosis development and progression.*

**Figure 5:**
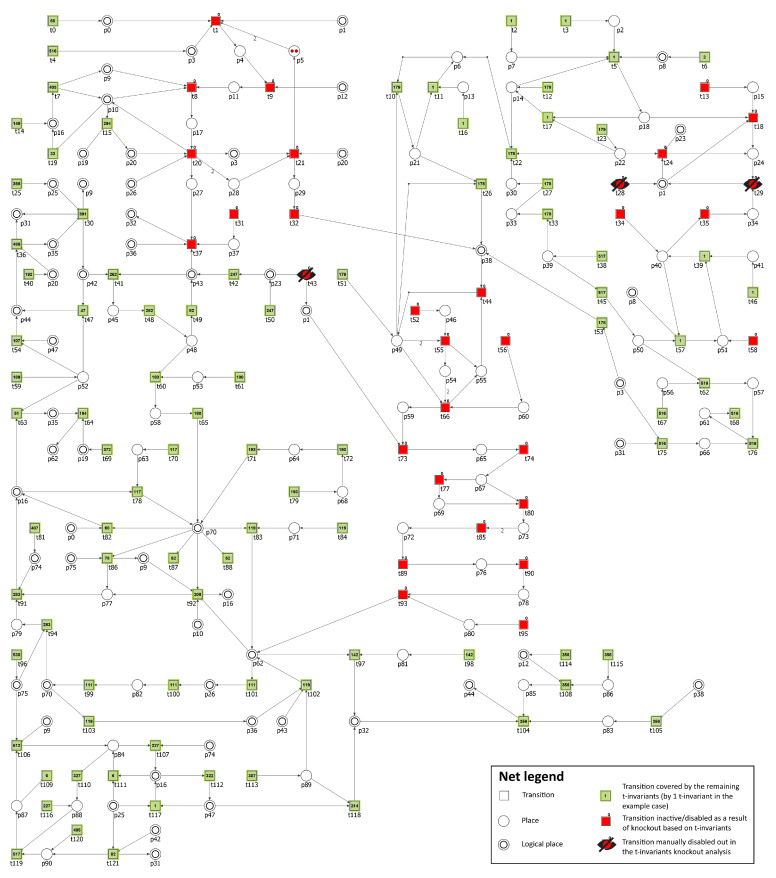
Graphical representation of the t-invariant based knockout impact of the following transitions: $t_{28}$, $t_{29}$, and $t_{43}$, on atherosclerosis ($t_{109}$) progression. The knocked-out transitions are denoted with crossed-out black circles. Transitions belonging to a support of any t-invariant are represented as filled green rectangles. The number inside the rectangle corresponds to the number of supports of t-invariants to which a given transition belongs. Transitions that do not belong to a support of any t-invariant are represented as red rectangles. The results have been obtained using Holmes software [76].

Since atherosclerosis is a complex vascular disease in which many processes and factors contribute to its development, acceleration, and progression, it has been decided to conduct several knockout experiments to investigate the influence of its most important factors. First, to model the effect of commonly used lipid-lowering, anti-hypercholesterolemic and anti-inflammatory drugs (statin) [80], HMG-CoA reductase activity ($p_{1}$) has been inhibited through excluding from the net the following transitions: $t_{28}$ (HMGCoA reductase activation), $t_{29}$ (reaction catalyzed by HMGCoA reductase phosphatase) and $t_{43}$ (increasing activity by SREBP2). As a result, it was possible to observe that cholesterol biosynthesis and transport of TAG within CM have been stopped. The number of t-invariants contributing to atherosclerosis progression dropped from 434 to 6 (see Table 7 and Figure 5).

Next, it has been analyzed the influence of the effect of the inhibition of Niemann–Pick C1-Like 1 (NPC1L1) protein ($p_{64}$) together with HMG-CoA reductase activity ($p_{1}$) through excluding from the model the following transitions: $t_{28}$ (HMGCoA reductase activation), $t_{29}$ (reaction catalyzed by HMGCoA reductase phosphatase), $t_{43}$ (increasing activity by SREBP2) and $t_{72}$ (NPC1L1 activation). In this way, it was possible to observe the result of adding to the statins therapy a drug called ezetimibe. Statin, inhibiting cholesterol synthesis, can upregulate cholesterol absorption. On the other hand, ezetimibe inhibits cholesterol absorption but can upregulate its synthesis. Nevertheless, such combined therapy has been proved to be an effective treatment choice with ezetimibe being one of few hypolipidemic drugs having atherosclerotic cardiovascular disease protective effect [42,91]. As a result of the conducted analysis, it was possible to observe that the number of t-invariants contributing to atherosclerosis progression dropped from 434 to 2 (see Table 7), which means that this process is strongly attenuated.

**Figure 6:**
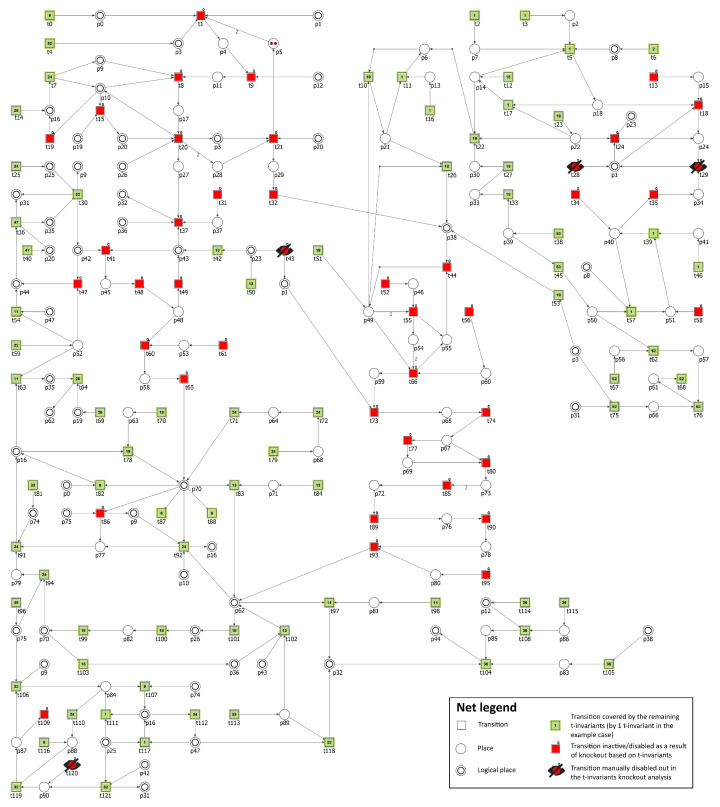
Graphical representation of the t-invariant based knockout impact of the following transitions: $t_{28}$, $t_{29}$, $t_{43}$, and $t_{120}$, on atherosclerosis ($t_{109}$) progression. The knocked-out transitions are denoted with crossed-out black circles. Transitions belonging to a support of any t-invariant are marked with filled green rectangles. The number inside the rectangle corresponds to the number of supports of t-invariants to which a given transition belongs. Transitions that do not belong to a support of any t-invariant are marked with red rectangles.The results were obtained using Holmes software [76].

The role of oxidative stress in atherosclerosis [96,98] has been also analyzed. To achieve this, transition $t_{120}$ (oxidation) has been excluded from the model and it turned out that there is no significant impact on the atherosclerosis progression (see Table 7). Therefore, the oxidative stress together with HMG-CoA reductase inhibition were attenuated, and as a result, atherosclerosis restraint was observed (see Table 7 and Figure 6). It is consistent with the literature, since it has been shown that antioxidant effect is one of the cholesterol-independent effects (pleiotropic) exerted by statins [99,100].

Finally, since there is evidence supporting a role of inflammation in the pathogenesis of atherosclerosis [101], the influence of drugs that target vascular inflammation has been explored. In order to do that, transition $t_{116}$ (influx of macrophages) has been excluded from the model. It turns out—similar to the case of oxidative stress alone—that there is no notable effect of such treatment (see Table 7). Therefore, the anti-inflammatory approach has been combined with HMG-CoA reductase inhibition, and as a result, it was possible to observe—similar as before—that the endogenous cholesterol synthesis and transport of TAG within CM have been stopped and the number of t-invariants contributing to atherosclerosis progression dropped from 434 to 5 (see Table 7).

As expected, the combination of anti-inflammatory and anti-oxidation treatment, together with HMG-CoA reductase blocking led to atherosclerosis inhibition (see Table 7).

In summary, the inhibition of HMG-CoA reductase itself, which is a crucial player in cholesterol synthesis and is often targeted by anti-hypercholesterolemic drugs, resulted in a slowdown of the development of atherosclerosis. Additionally, in order to observe the results of the therapy involving the administration of opposing drugs, we inhibited also Niemann–Pick C1-Like 1 (NPC1L1) protein, which is essential for intestinal cholesterol absorption. As a result we could observe that the atherosclerosis progression was strongly attenuated, but still not stopped. Another combination that we analyzed, simulating antioxidant effects exerted by statins, was the inhibition of oxidative stress together with HMG-CoA reductase activity which led finally to atherosclerosis restraint. In the above-mentioned knockout experiments, HDL-mediated processes stayed unaffected.

**Scenario** **4.** *The influence of the microsomal triglyceride transfer protein (MTTP) inhibition on the atherosclerosis development and progression.*

Microsomal triglyceride transfer protein (MTTP) is a member of a protein group that is able to transfer lipids between membranes and it plays an essential role in lipids metabolism. It is involved in the biogenesis of very low-density lipoproteins (VLDLs) and chylomicrons (CMs) through the transfer of neutral lipids and the assembly of APOB-containing lipoproteins. The inhibition of MTTP blocks the hepatic secretion of VLDL and intestinal secretion of CM being the promising therapeutic target for lowering of low-density lipoprotein (LDL) and what follows, causing atherosclerotic plaque regression [89,102,103]. Moreover, it was also shown that the inhibitors of intestinal MTTP can lower triglyceride without causing hepatic steatosis [42].

**Figure 7:**
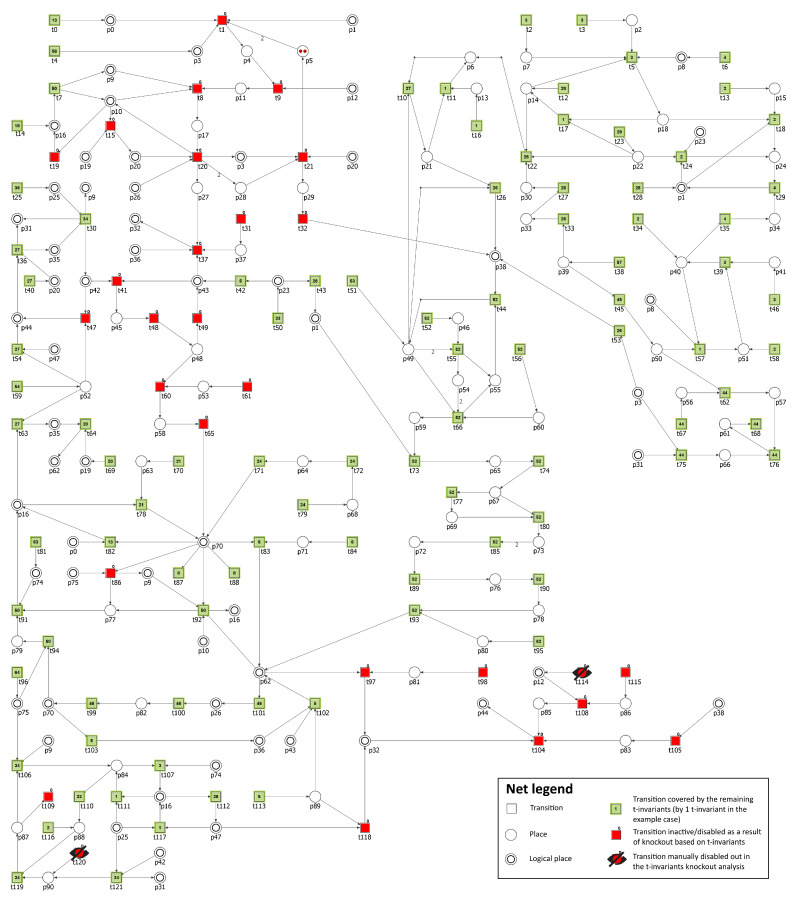
Graphical representation of the t-invariant based knockout impact of the following transitions: $t_{114}$ and $t_{120}$, on atherosclerosis ($t_{109}$) progression. The knocked-out transitions are denoted with crossed-out black circles. Transitions belonging to a support of any t-invariant are represented as filled green rectangles. The number inside the rectangle corresponds to the number of supports of t-invariants to which a given transition belongs. Transitions that do not belong to a support of any t-invariant are represented as red rectangles.The results have been obtained using Holmes software [76].

In order to examine the effect of MTTP inhibition, transition $t_{114}$ (MTTP synthesis) has been excluded from the studied model. Analyzing the obtained results, it was noticed that the number of t-invariants contributing to atherosclerosis progression dropped from 434 to 22 (see Table 7). Since the oxidation stress turned out to be the most important remaining factor accelerating atherosclerosis, transition $t_{120}$ (oxidation) was additionally knocked-out from the model, and the atherosclerotic progression was halted (see Table 7 and Figure 7).

In summary, the inhibition of MTTP, which is involved in lipid transfer and metabolism, and is also a promising therapeutic target for lowering of low density lipoprotein (LDL), caused the decrease in atherosclerosis progression. However, the additional blockade of oxidative stress resulted in the complete atherosclerotic development attenuation.

These finding are consistent with the results observed in [102]. Research on the use of such a blocker is ongoing. Recently, the drug lomitapide (an MTTP inhibitor), approved in Europe for treatment, has been used to lower the cholesterol levels associated with homozygous familial hypercholesterolemia, reducing the risk of cardiovascular events, such as myocardial infarction and stroke.

**Scenario** **5.** *The influence of the Acyl-CoA:cholesterol acyltransferase (ACAT) inhibition on the atherosclerosis development and progression.*

Acyl-CoA:cholesterol acyltransferase (ACAT) is a cytoplasmic enzyme responsible for cholesterol esterification, playing a central role in regulating intracellular free cholesterol levels in humans. The foam cells that are formed in atherosclerosis, contain great quantities of cholesteryl esters, whose presence is directly related to ACAT activity. It is the reason why ACAT inhibitors are considered as a potential antiatherosclerotic drugs. In human trials, ACAT inhibitors have been tested as supplements to statins and shown no significant efficacy in reducing plaque volume. Nevertheless, these trials have not examined if ACAT inhibitors stabilize plaques and have been eventually considered inconclusive [95].

Here, in order to evaluate the knockout impact of ACAT on atherosclerosis, transitions $t_{0}$ (ACAT activation in the intestinum) and $t_{98}$ (ACAT activation in the liver) have been excluded from the studied system. As a result of the conducted analysis, it has been observed that the number of t-invariants contributing to atherosclerosis progression dropped from 434 to 17 (see Table 7).

It has been shown in [95] that global inhibition of ACAT can result in many of the undesirable side effects; moreover, ACAT has been inhibited locally, in liver and intestinum separately. This way, many side effects can be avoided. In the case of transition $t_{98}$ (ACAT activation in the liver) being knocked-out from the model, the number of t-invariants contributing to atherosclerosis progression dropped from 434 to 308 (see Table 7), while in the situation of transition $t_{0}$ (ACAT activation in the intestine) inhibition this number decreased from 434 to 25 (see Table 7). Moreover, in both cases, transition $t_{120}$ (oxidation) was excluded from the net, inhibiting, in that way, the oxidative stress. It turned out that there is no significant impact on the atherosclerosis progression in the case of ACAT located in the liver, but for ACAT located in the intestine, the development of atherosclerosis has been significantly suppressed (see Table 7).

In summary, the inhibition of ATAC located in the interstitium, together with oxidative stress, has a great influence on the atherosclerosis development and progression.

**Scenario** **6.** *Analysis of other factors influencing atherosclerosis progression.*

Other factors that seem to influence atherosclerosis by reducing the number of processes represented by t-invariants where $t_{109}$ is present are $t_{73}$ (mevalonate synthesis), which in turn disable whole $m_{1}$ (endogenous synthesis of cholesterol mainly in the liver), $t_{113}$ (SRB1 expression) and $t_{51}$ (acetyl-CoA synthesis from glucose in the liver). In particular, the first and the latter reduce both overall number of t-invariants by respectively 51.1% (100–48.9%) and 56.2% (100–43.8%) and the processes where atherosclerosis is present by respectively 54.0% (100–46%) and 54.9% (100–45.1%) as can be seen in Table 7. In summary, one can observe that it is rather difficult to significantly reduce atherosclerosis processes while at the same time leaving other processes intact. One example of such an action can be a knockout of transition $t_{0}$ (inhibition of ACAT in the intestine), which reduces atherosclerosis processes to 5.17%, while approximately only 28% t-invariants remains.

### 3.3. The Knockout Analysis Based on Simulation

The last type of analysis involved simulation knockout. In such an analysis some specific transitions have been set as disabled, but this time, the impact of such an action on the atherosclerosis process (transition $t_{109}$) was computed by performing a simulation. Every simulation involved disabling different transitions and gathering data about transitions firing in 350,000 steps. Such a simulation has been repeated 50 times and the average transition firings and tokens accumulations have been collected. In this type of simulation, every active transition has a 50% chance of firing, and the analyzed transition $t_{109}$ in a simulation when nothing has been knocked-out fired in 27.29% of all simulation steps. The results are given in Table 8. In this table, only those results that indicate significant changes in the atherosclerosis process are included.

**Figure 8:**
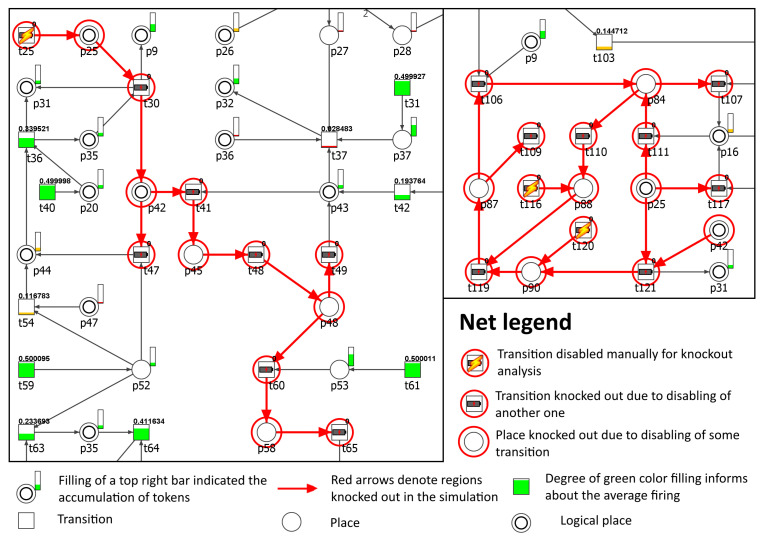
Graphical representation of the knockout impact of the following transitions: $t_{25}$, $t_{116}$ and $t_{120}$, on atherosclerosis ($t_{109}$) progression. Inactive transitions, according to the simulation knockout, are marked with red circles. Active transitions are represented as rectangles filled with green or yellow color, which indicates whether the activity of a given transition has decreased (partially filled) or stayed intact (fully filled) as compared to the reference set. The results have been obtained using Holmes software [76].

As one can see, transitions $t_{116}$, $t_{120}$, attenuation of inflammation and/or oxidative stress and HMG-CoA reductase have all the greatest impact on the production of tokens by $t_{109}$ (atherosclerosis). More subtle influence on the reduction of the atherosclerotic process comes from transition $t_{25}$ (LIPC activation process). If two transitions will be knocked-out: $t_{116}$ and $t_{120}$, the atherosclerosis progression within the analyzed model will be disabled completely. Such a scenario is presented in Figure 8, where all paths disabled by these transitions knockout are presented. Finally, it should be noted that the simulation has been performed on the non-stochastic, classical Petri net, using simulation parameters explained in the Methods section. A potentially valuable extension to this approach would be to prepare and study a stochastic Petri net of the cholesterol metabolism and atherosclerosis progression. Simulation of such a net could possibly reveal a more subtle and detailed influence of the knocked-out processes on the rest of the net.

## 4. Conclusions

This study has revealed that controlling cholesterol metabolism by blocking selected pathways cannot stop atherosclerotic plaque formation in the proposed model completely.

Moreover, it has been shown that inhibition of the action of key cholesterol metabolism players, such as (1) HMG-CoA reductase together with the protein NPC1L1; (2) HMG-CoA reductase and the local inflammatory process; (3) HMG-CoA reductase alone; and (4) MTTP significantly reduced the development of atherosclerotic plaque in the studied model.

Moreover, in the study, we discovered that combining different classes of drugs targeting significant components of cholesterol metabolism, namely (1) HMG-CoA reductase, (2) MTTP, or (3) intestinal ACAT, along with blocking the impact of oxidative stress, made it possible to control the development and progression of atherosclerosis in the proposed model.

## Figures and Tables

**Table 1:** The list of places of the model.

Place	Biological Meaning	References	Place	Biological Meaning	References
$p_{0}$	ACAT in the intestine	[78]	$p_{46}$	thiolase	[79]
$p_{1}$	HMGCoA reductase active	[80]	$p_{47}$	HDL2	[81,82]
$p_{2}$	CaMKK beta	[83]	$p_{48}$	LDL cholesterol as CE in endosome	[84,85]
$p_{3}$	Free fatty acids (FFA) in intestinal lumen in micelles	[78]	$p_{49}$	Acetyl-CoA	[28,79,86]
$p_{4}$	Nascent chylomicrons (CM) with APOB48	[78]	$p_{50}$	cAMP PKA activated	[83]
$p_{5}$	TAG in enterocytes	[78]	$p_{51}$	Phosphoprotein phosphatase inhibitor 1 PPI1 with an increase activity	[87]
$p_{6}$	ACC activated	[83,86]	$p_{52}$	CE transfer protein CETP in blood	[82]
$p_{7}$	LKB1 serine threonine kinase 1	[83]	$p_{53}$	Lysosomal lipases	[84,88]
$p_{8}$	Phosphoprotein phosphatase with a decrease activity	[83]	$p_{54}$	Acetoacetyl-CoA	[28,79]
$p_{9}$	APOE	[88]	$p_{55}$	CoA	[28,79]
$p_{10}$	APOC2	[81,88]	$p_{56}$	Hormone sensitive lipase (HSL)	[87]
$p_{11}$	Nascent CM in the blood	[78]	$p_{57}$	Hormone sensitive lipase HSL phosphorylated	[87]
$p_{12}$	MTTP	[42,89]	$p_{58}$	Free cholesterol in endosome in intestinum	[78]
$p_{13}$	MCD	[86]	$p_{59}$	HMG CoA	[80]
$p_{14}$	AMP activated protein kinase OH AMPK inactive	[83]	$p_{60}$	HMG CoA synthase	[80]
$p_{15}$	HMG-CoA reductase phosphatase with a decrease activity	[80]	$p_{61}$	Free fatty acids FFA in adipose tissues	[87]
$p_{16}$	HDL3 cholesterol CE in blood	[81]	$p_{62}$	High unesterified cholesterol pool in the liver	[28,78]
$p_{17}$	Mature CM with APOB48, APOC2, APOE	[88]	$p_{63}$	Hydrolase of cholesterol esters	[90]
$p_{18}$	AMP activated protein kinase AMPK active	[83]	$p_{64}$	NPC1L1	[42,91]
$p_{19}$	LRP1	[88,92]	$p_{65}$	Mevalonate	[28]
$p_{20}$	Lipoprotein lipase (LPL)	[88]	$p_{66}$	Stored TAG	[88]
$p_{21}$	Malonyl CoA increases	[86]	$p_{67}$	Isopentenyl PP	[28]
$p_{22}$	Protein phosphatase 2C with an increase activity	[83]	$p_{68}$	Low cholesterol in diet	[78]
$p_{23}$	Low free cholesterol pool in intestinum and in the peripheral tissues	[78,84]	$p_{69}$	Geranyl PP	[28]
$p_{24}$	HMG-CoA reductase phosphorylated inactive	[80]	$p_{70}$	High free cholesterol pool in intestinum	[85,90,93]
$p_{25}$	LIPC hepatic lipase	[84,94]	$p_{71}$	Apical sodium bile acid transporter ASBT	[78]
$p_{26}$	Bile acids	[28]	$p_{72}$	Squalene	[28]
$p_{27}$	Remnant CM with APOB48 APOE	[88]	$p_{73}$	Farnesyl PP	[28]
$p_{28}$	MAG in intestinal lumen in micelles	[78]	$p_{74}$	LCAT	[81]
$p_{29}$	FFA and MAG in enterocytes	[78]	$p_{75}$	ABCA1 cholesterol efflux regulatory protein CERP	[93]
$p_{30}$	cAMP PKA low activated	[83]	$p_{76}$	2,3-oxidosqualene	[28]
$p_{31}$	Free fatty acids FFA	[87,94]	$p_{77}$	HDL cholesterol non-CE	[81]
$p_{32}$	Cholesterol stored as cholesteryl esters in the liver	[95]	$p_{78}$	Lanosterol	[28]
$p_{33}$	Low cAMP	[83]	$p_{79}$	Cholesterol from enterocytes and peripheral tissues transported to the blood	[78,93]
$p_{34}$	HMG-CoA reductase phosphatase	[80]	$p_{80}$	Enzymes in ER membranes	[28]
$p_{35}$	IDL	[82,85]	$p_{81}$	ACAT in the liver	[95]
$p_{36}$	Remnant CM receptors in the liver	[85]	$p_{82}$	Biliary cholesterol	[28]
$p_{37}$	LDL receptor related protein	[84,85]	$p_{83}$	TAG synthesized in the liver	[85]
$p_{38}$	Increased FA in the liver	[85,86]	$p_{84}$	Nascent HDL	[81]
$p_{39}$	cAMP	[83]	$p_{85}$	MTTP APOB-100 complex	[42,89]
$p_{40}$	PPI 1 OH	[87]	$p_{86}$	APOB-100	[42,89]
$p_{41}$	Phosphoprotein phosphatase with an increase activity	[87]	$p_{87}$	Foamy cells	[88]
$p_{42}$	LDL cholesterol in serum	[84]	$p_{88}$	Macrophages	[88]
$p_{43}$	High expression of LDLR on cell membrane	[84]	$p_{89}$	SRB1	[84]
$p_{44}$	Nascent VLDL reach in TAG secreted from the liver into the blood	[85]	$p_{90}$	Small dense LDL	[84]
$p_{45}$	LDLR–LDL complex	[84]			

**Table 2:** The list of transitions of the model.

Transition	Biological Meaning	References	Transition	Biological Meaning	References
$t_{0}$	ACAT activation in the intestine	[95]	$t_{61}$	Lysosomal lipases activation	[84]
$t_{1}$	Nascent CM synthesis in enterocytes	[78]	$t_{62}$	Phosphorylation by PKA	[83,87,95]
$t_{2}$	LKB1 activation	[83]	$t_{63}$	Conversion HDL into IDL	[82,85]
$t_{3}$	Processes increasing intracellular calcium	[83]	$t_{64}$	Activation by LRP1	[92]
$t_{4}$	Diet and hypertension	[78,85]	$t_{65}$	Free cholesterol effluxes endosome	[84]
$t_{5}$	AMP activated protein kinase AMPK phosphorylation	[83]	$t_{66}$	Acetyl-CoAs conversion	[28,79]
$t_{6}$	Processes decreasing phosphoprotein phosphatase	[83]	$t_{67}$	HSL activation	[87]
$t_{7}$	Exchanging HDL components in blood	[81]	$t_{68}$	FFA pool in adipose tissue increases	[87,94]
$t_{8}$	Nascent CM exchange components with HDL	[81]	$t_{69}$	LRP1 synthesis	[92]
$t_{9}$	Transport mainly TAG within nascent chylomicrons from the intestine to the blood	[78]	$t_{70}$	Hydrolase of cholesterol esters activation	[90]
$t_{10}$	Carboxylation catalysed by acetyl-CoA carboxylase ACC	[28,86]	$t_{71}$	Cholesterol transport from the lumen to the intestine	[78]
$t_{11}$	Decarboxylation	[86]	$t_{72}$	NPC1L1 activation	[42,91]
$t_{12}$	Processes increasing AMP activated protein kinase 0H AMPK inactive	[83]	$t_{73}$	Mevalonate synthesis	[28]
$t_{13}$	Processes decreasing HMGCoA reductase phosphatase activity	[28,80]	$t_{74}$	Reaction phosphorylation catalysed by ATP	[28]
$t_{14}$	HDL synthesis in the liver	[81]	$t_{75}$	TAG storage in adipocytes	[87]
$t_{15}$	LPL activation	[88]	$t_{76}$	Hydrolysis of stored TAG	[87,88]
$t_{16}$	Malonyl CoA decarboxylase MCD activation	[86]	$t_{77}$	Reaction condensation	[28]
$t_{17}$	Dephosphorylation by protein phosphatase 2C	[83]	$t_{78}$	Conversion from CE found in HDL into free cholesterol pool	[81,90]
$t_{18}$	HMG-CoA reductase inactivation by phosphorylation	[28,80,83]	$t_{79}$	Processes lowering cholesterol	[78]
$t_{19}$	APOC2 returned to HDL cholesterol	[81,88]	$t_{80}$	Reaction forming farnesyl PP	[28]
$t_{20}$	TAG distribution from CM	[78,88]	$t_{81}$	LCAT activation in serum	[81]
$t_{21}$	FFA absorption in enterocyte	[78]	$t_{82}$	Processes catalyzed by ACAT	[95]
$t_{22}$	Dephosphorylation of ACC and its activation	[83]	$t_{83}$	Reabsorption in the intestine and return to the liver	[78]
$t_{23}$	Protein phosphatase activation	[83]	$t_{84}$	ASBT activation	[78]
$t_{24}$	Dephosphorylation	[78,83,84]	$t_{85}$	Reaction catalyzed by squalene synthase	[28]
$t_{25}$	LIPC activation	[84,94]	$t_{86}$	Efflux of cholesterol to APOA1 and APOE catalyzed by ABCA1	[88]
$t_{26}$	FA synthesis in the liver	[85,86]	$t_{87}$	Steroid synthesis	[85,90,93]
$t_{27}$	Decreased PKA activation	[83]	$t_{88}$	Remaining cholesterol removed by fecal sterols	[85,90,93]
$t_{28}$	HMG-CoA reductase activation	[28,80]	$t_{89}$	Reaction catalysed by squalene monooxygenase	[28]
$t_{29}$	Reaction catalyzed by HMG-CoA reductase phosphatase	[28,80]	$t_{90}$	Reaction catalyzed by squalene epoxidase	[28]
$t_{30}$	Conversion into LDL	[84]	$t_{91}$	Conversion cholesterol into CE	[78,81,93]
$t_{31}$	LDLR synthesis	[84]	$t_{92}$	HDL secreted by enterocytes and by the liver	[78]
$t_{32}$	Binding with glycerol albumin	[78,85]	$t_{93}$	Reaction 19 leading to cholesterol synthesis in liver	[28]
$t_{33}$	Processes decreasing cAMP	[83]	$t_{94}$	Transport by ABCA1	[93]
$t_{34}$	PPI 1 OH activation	[87]	$t_{95}$	Enzymes activation	[28]
$t_{35}$	HMGCoA reductase phosphatase activation	[28,80]	$t_{96}$	ABCA1 synthesis	[93]
$t_{36}$	Conversion VLDL into IDL TAG hydrolysis	[85]	$t_{97}$	Re-esterification of cholesterol by ACAT in the liver	[95]
$t_{37}$	CM endocytosis in the liver	[84]	$t_{98}$	ACAT activation in the liver	[95]
$t_{38}$	Hormonal processes increasing cAMP	[83]	$t_{99}$	Cholesterol pool increases in the intestinum because of biliary cholesterol	[28]
$t_{39}$	Reaction catalyzed by phosphoprotein phosphatase	[87]	$t_{100}$	Formation of the biliary cholesterol	[28]
$t_{40}$	Pancreatic synthesis	[88]	$t_{101}$	Bile acids synthesis	[28]
$t_{41}$	Binding LDL and LDLR	[84]	$t_{102}$	Reaction increasing cholesterol pool in the liver via RME	[84]
$t_{42}$	LDLR expression on cell membrane	[85]	$t_{103}$	Expression remnant CE receptors in the liver when intestinal pool is high	[85]
$t_{43}$	Increasing activity by SREBP2	[78,80,84]	$t_{104}$	Reaction forming nascent VLDL reach in TAG in the liver	[85]
$t_{44}$	Beta oxidation	[28,79,85,86]	$t_{105}$	TAG synthesis in the liver	[85,86]
$t_{45}$	Increased PKA activation	[83]	$t_{106}$	Efflux of free cholesterol from peripheral tissues	[93]
$t_{46}$	Phosphoprotein phosphatase activation	[87]	$t_{107}$	Conversion nascent HDL into HDL3	[81]
$t_{47}$	CE transfer from LDL	[84]	$t_{108}$	Forming complex	[42,89]
$t_{48}$	Endocytosis via RME	[84]	$t_{109}$	Atherosclerosis	[88]
$t_{49}$	Receptor being returned stimulated by lower pH	[84]	$t_{110}$	Transport into peripheral tissue	[81,88]
$t_{50}$	Processes lowering free cholesterol pool in intestine and in the peripheral tissues	[78,84]	$t_{111}$	Conversion HDL3 into nascent LDL	[81]
$t_{51}$	acetyl-CoA synthesis from glucose in the liver	[28,79]	$t_{112}$	Conversion HDL3 into HDL2	[81]
$t_{52}$	Thiolase activation	[79]	$t_{113}$	SRB1 expression	[84]
$t_{53}$	Internalized from blood by the liver	[78,85,86]	$t_{114}$	MTTP synthesis	[42,89]
$t_{54}$	CE transfer from HDL2	[81,82]	$t_{115}$	APOB100 synthesis in the liver and secreted into circulation	[42,89]
$t_{55}$	Reaction catalyzed by thiolase	[79]	$t_{116}$	Influx of macrophages	[88]
$t_{56}$	HMGCoA synthase activation in cytoplasm	[28,80]	$t_{117}$	Conversion HDL2 into HDL3	[81]
$t_{57}$	High PPI OH phosphorylation	[87]	$t_{118}$	Cholesterol CE transport to the liver	[84]
$t_{58}$	Processes increasing PPI1 activity	[87]	$t_{119}$	Binding with SRA-2 on macrophages	[84,88]
$t_{59}$	CETP secretion from the liver	[82]	$t_{120}$	Oxidation	[96]
$t_{60}$	CE hydrolysis	[84]	$t_{121}$	Degradation	[84]

**Table 3:** The MCT sets of the model and their biological interpretations.

MCT-Set	Contained Transitions	Biological Interpretation
$m_{1}$	$t_{44}$, $t_{52}$, $t_{55}$, $t_{56}$, $t_{66}$, $t_{73}$, $t_{74}$, $t_{77}$, $t_{80}$, $t_{85}$, $t_{89}$, $t_{90}$, $t_{93}$, $t_{95}$	Endogenous synthesis of cholesterol mainly in the liver
$m_{2}$	$t_{1}$, $t_{8}$, $t_{9}$, $t_{20}$, $t_{21}$, $t_{31}$, $t_{32}$, $t_{37}$	Transport of TAG within CM
$m_{3}$	$t_{62}$, $t_{67}$, $t_{68}$, $t_{75}$, $t_{76}$	Transport of TAG within VLDL
$m_{4}$	$t_{104}$, $t_{105}$, $t_{108}$, $t_{114}$, $t_{115}$	Lipolysis
$m_{5}$	$t_{22}$, $t_{26}$, $t_{27}$, $t_{33}$	Increase in fatty acids synthesis
$m_{6}$	$t_{2}$, $t_{3}$, $t_{5}$	Regulation of AMPK activity
$m_{7}$	$t_{60}$, $t_{61}$, $t_{65}$	Cholesterol influx into cells through uptake and CE hydrolysis found in LDL
$m_{8}$	$t_{71}$, $t_{72}$, $t_{79}$	Intracellular transport of cholesterol
$m_{9}$	$t_{11}$, $t_{16}$	Entry of LDL into the cells
$m_{10}$	$t_{13}$, $t_{18}$	Regulation of HMG-CoA activity
$m_{11}$	$t_{29}$, $t_{35}$	Increase in the free cholesterol pool via the CE coming from HDL
$m_{12}$	$t_{39}$, $t_{46}$	Increase in CE in HDL cholesterol in the serum
$m_{13}$	$t_{41}$, $t_{48}$	The bile acids biosynthesis from cholesterol
$m_{14}$	$t_{70}$, $t_{78}$	Regulation of ACAT activity
$m_{15}$	$t_{83}$, $t_{84}$	ACC activation by malonyl-CoA decarboxylation
$m_{16}$	$t_{91}$, $t_{94}$	Reverse transport of bile acids to the liver
$m_{17}$	$t_{97}$, $t_{98}$	PPA activity regulation
$m_{18}$	$t_{99}$, $t_{100}$	HMG-CoA reductase phosphatase activity

**Table 4:** The impact of a knockout of selected net elements (MCT sets or single transitions) depending on the percentage of affected transitions calculated on the basis of both simulation knockout and the approach described in [77], identically as in [97].

Knocked-out MCT Set	Biological Function	Affected Transitions
$t_{38}$	Hormonal processes increasing cAMP	33.6%
$t_{4}$	Increased PKA activation	29.5%
$t_{45}$	Diet and hypertension	29.5%
$m_{3}$	Transport of TAG within VLDL	27.9% (23.8%)
$t_{51}$	Acetyl-CoA synthesis from glucose in the liver	18.3%
$t_{36}$	Conversion VLDL into IDL TAG hydrolysis	17.2%
$t_{50}$	Processes lowering free cholesterol pool in intestine and in the peripheral tissues	13.9%
$m_{4}$	Lipolysis	13.1% (9.0%)
$m_{1}$	Endogenous synthesis of cholesterol mainly in the liver	11.5% (0.0%)
$t_{42}$	LDLR expression on cell membrane	11.5%
$t_{25}$	LIPC activation	9.8%
$t_{101}$	Bile acids synthesis	9.0%
$t_{103}$	Expression remnant CE receptors in the liver when intestinal pool is high	8.2%
$t_{0}$	ACAT activation in the intestine	8.2%
$t_{30}$	Conversion into LDL	7.4%
$m_{2}$	Transport of TAG within CM	6.6% (0.0%)
$t_{6}$	Processes decreasing phosphoprotein phosphatase	6.6%
$m_{11}$	Increase in the pool of free cholesterol pool via the CE coming from HDL	5.7%
$t_{10}$	Carboxylation catalyzed by acetyl-CoA carboxylase (ACC)	5.7%
$t_{12}$	Processes increasing AMP activated protein kinase 0H AMPK inactive	5.7%
$t_{23}$	Protein phosphatase activation	5.7%
$t_{119}$	Binding with SRA-2 on macrophages	5.7%
$m_{6}$	Regulation of AMPK activity	4.9% (3.3%)
$m_{13}$	The bile acids biosynthesis from cholesterol	4.9% (3.3%)
$t_{81}$	LCAT activation in serum	4.9%
$t_{96}$	ABCA1 synthesis	4.9%
$m_{5}$	Increase in fatty acids synthesis	3.3% (0.0%)
$m_{12}$	Increase in CE in HDL cholesterol in the serum	3.3% (1.6%)
$m_{16}$	Reverse transport of bile acids to the liver	3.3% (1.6%)
$t_{7}$	Exchanging HDL components in blood	3.3%
$t_{59}$	CETP secretion from the liver	3.3%
$t_{112}$	Conversion HDL3 into HDL2	3.3%
$m_{7}$	Cholesterol influx into cells through uptake and CE hydrolysis found in LDL	2.46% (0%)
$m_{8}$	Intracellular transport of cholesterol	2.46% (0%)
$t_{69}$	LRP1 synthesis	2.46%
$t_{113}$	SRB1 expression	2.46%

**Table 5:** Important cholesterol molecules within the model.

Molecule	Associated Places
LDL	$p_{37}$ (LDL receptor related protein), $p_{42}$ (LDL cholesterol in serum), $p_{45}$ (LDLR–LDL complex), $p_{48}$ (LDL cholesterol as CE in endosome), $p_{90}$ (small dense LDL)
VLDL	$p_{44}$ (nascent VLDL reach in TAG secreted from the liver into the blood)
IDL	$p_{35}$ (IDL)
HDL	$p_{16}$ (HDL3 cholesterol CE in blood), $p_{47}$ (HDL2), $p_{77}$ (HDL cholesterol non-CE), $p_{84}$ (nascent HDL)
CM	$p_{4}$ (nascent chylomicrons (CM) with APOB48), $p_{11}$ (nascent CM in the blood), $p_{36}$ (remnant CM receptors in the liver)

**Table 6:** Knockout impact of selected transitions responsible for cholesterol particle production on MCT sets and t-invariants.

Molecule	Knocked-Out Transitions	Disabled Transitions and MCT Sets	Number of Remaining t-Invariants (Percentage of Remaining t-Invariants)	Number of Remaining t-Invariants Which Contain Transition $t_{109}$ (Atherosclerosis) in Their Supports (Percentage of Remaining t-Invariants Which Contain Transition $t_{109}$ (Atherosclerosis) in Their Supports
LDL	$t_{30}$, $t_{31}$, $t_{41}$, $t_{120}$, $t_{121}$	$m_{2}$, $m_{7}$, $m_{13}$, $t_{15}$, $t_{19}$, $t_{47}$, $t_{49}$, $t_{86}$, $t_{106}$, $t_{109}$, $t_{110}$, $t_{116}$, $t_{119}$	78 (out of 3871) (2%)	0 (out of 434) (0%)
VLDL	$t_{47}$, $t_{54}$, $t_{104}$	$m_{2}$, $m_{4}$, $m_{17}$, $t_{15}$, $t_{36}$, $t_{40}$, $t_{118}$	185 (out of 3871) (4.8%)	22 (out of 434) (5%)
IDL	$t_{36}$, $t_{63}$	$m_{2}$, $m_{3}$, $m_{4}$, $m_{7}$, $m_{13}$, $m_{17}$, $t_{15}$, $t_{30}$, $t_{40}$, $t_{47}$, $t_{49}$, $t_{54}$, $t_{59}$, $t_{63}$, $t_{64}$, $t_{69}$, $t_{118}$, $t_{121}$	78 (out of 3871) (2%)	22 (out of 434) (5%)
HDL	$t_{14}$, $t_{19}$, $t_{82}$, $t_{86}$, $t_{91}$, $t_{92}$, $t_{106}$, $t_{107}$, $t_{111}$, $t_{112}$, $t_{117}$	$m_{14}$, $t_{7}$, $t_{15}$, $t_{54}$, $t_{63}$, $t_{81}$, $t_{94}$, $t_{96}$, $t_{110}$, $t_{118}$	80 (out of 3871) (2%)	19 (out of 434) (4.3%)
CM	$t_{1}$, $t_{9}$, $t_{103}$	$m_{2}$, $t_{102}$	1136 (out of 3871) (29%)	33 (out of 434) (7.5%)

**Table 7:** Knockout impact of selected transitions corresponding to known factors responsible for atherosclerosis progression on MCT sets and t-invariants.

Molecule	Knocked-Out Transitions	Disabled Transitions and MCT Sets	Number of Remaining t-Invariants (Percentage of Remaining t-Invariants)	Number of Remaining t-Invariants Which Contain Transition $t_{109}$ (Atherosclerosis) in Their Supports (Percentage of Remaining t-Invariants Which Contain Transition $t_{109}$ (Atherosclerosis) in Their Supports)
Inhibition of HMG-CoA reductase	$m_{11}$, $t_{28}$, $t_{43}$	$m_{1}$, $m_{2}$, $m_{10}$, $t_{24}$, $t_{34}$	559 (14.5%)	6 (out of 434) (1.4%)
Inhibition of HMG-CoA reductase and the Niemann–Pick C1-Like 1 (NPC1L1) protein	$m_{8}$, $m_{11}$, $t_{28}$, $t_{43}$	$m_{1}$, $m_{2}$, $m_{10}$, $t_{24}$, $t_{34}$	366 (out of 3871) (9.5%)	2 (out of 434) (0.05%)
Attenuation of oxidative stress	$t_{120}$	-	1524 (out of 3871) (39.3%)	378 (out of 434) (87%)
Attenuation of oxidative stress and HMG-CoA reductase	$m_{11}$, $t_{28}$, $t_{43}$, $t_{120}$	$m_{1}$, $m_{2}$, $m_{7}$, $m_{10}$, $m_{13}$, $t_{15}$, $t_{19}$, $t_{47}$, $t_{49}$, $t_{58}$, $t_{86}$, $t_{109}$	74 (out of 3871) (2%)	0 (out of 434) (0%)
Attenuation of inflammation	$t_{116}$	-	2358 (out of 3871) (60.9%)	275 (out of 434) (63.3%)
Attenuation of inflammation and HMG-CoA reductase	$m_{11}$, $t_{28}$, $t_{43}$, $t_{116}$	$m_{1}$, $m_{2}$, $m_{10}$, $t_{24}$, $t_{34}$, $t_{58}$	332 (out of 3871) (8.5%)	5 (out of 434) (1%)
Attenuation of inflammation, oxidative stress and HMG-CoA reductase	$m_{11}$, $t_{28}$, $t_{43}$, $t_{116}$, $t_{120}$	$m_{1}$, $m_{2}$, $m_{7}$, $m_{10}$, $m_{13}$, $t_{15}$, $t_{19}$, $t_{24}$, $t_{34}$, $t_{47}$, $t_{49}$, $t_{58}$, $t_{86}$, $t_{109}$	66 (out of 3871) (1.7%)	0 (out of 434) (0%)
Inhibition of microsomal triglyceride transfer protein (MTTP)	$t_{114}$	$m_{2}$, $m_{5}$, $m_{17}$	483 (out of 3871) (12.5%)	22 (out of 434) (5%)
Inhibition MTTP and oxidative stress	$t_{114}$, $t_{120}$	$m_{2}$, $m_{4}$, $m_{5}$, $m_{7}$, $m_{13}$, $m_{17}$, $t_{15}$, $t_{19}$, $t_{47}$, $t_{49}$, $t_{86}$, $t_{109}$, $t_{118}$	94 (out of 3871) (2.4%)	0 (out of 434) (0%)
Inhibition of Acyl-CoA: cholesterol acyltransferase (ACAT) in the liver	$m_{17}$	-	2785 (out of 3871) (71.95%)	308 (out of 434) (70.97%)
Inhibition of ACAT in the intestine	$t_{0}$	$m_{2}$, $t_{82}$	1103 (out of 3871) (28.49%)	25 (out of 434) (5.17%)
Inhibition of ACAT both in the liver and intestine	$m_{17}$, $t_{0}$	$m_{2}$, $t_{82}$	731 (out of 3871) (18.9%)	17 (out of 434) (3.9%)
Inhibition of ACAT in the intestine and oxidative stress	$t_{0}$, $t_{120}$	$m_{2}$, $m_{7}$, $m_{13}$, $t_{15}$, $t_{19}$, $t_{47}$, $t_{49}$, $t_{82}$, $t_{86}$, $t_{109}$	141 (out of 3871) (3.64%)	0 (out of 434) (0%)
Other discovered factors influencing atherosclerosis progression				
Inhibition of AMP activated protein kinase OH AMPK	$t_{12}$	$m_{5}$, $m_{10}$	3092 (out of 3871) (79.8%)	384 (out of 434) (88.4%)
Inhibition of mevalonate synthesis	$m_{1}$		1894 (out of 3871) (48.9%)	200 (out of 434) (46.0%)
Inhibition of SRB1 synthesis	$t_{113}$	$t_{102}$, $t_{118}$	2333 (out of 3871) (60.2%)	293 (out of 434) (67.5%)
Inhibition of mevalonate and SRB1	$t_{73}$, $t_{113}$	$m_{1}$, $t_{102}$, $t_{118}$	682 (out of 3871) (17.6%)	79 (out of 434) (18.2%)
Inhibition of HMG-CoA reductase, mevalonate, SRB1	$m_{11}$, $t_{28}$, $t_{43}$, $t_{73}$, $t_{113}$	$m_{2}$, $m_{10}$, $t_{24}$, $t_{34}$, $t_{58}$, $t_{103}$, $t_{102}$, $t_{118}$	474 (out of 3871) (12.2%)	42 (out of 434) (9.6%)
Inhibition of acetyl-CoA synthesis from glucose in the liver	$t_{51}$	$m_{1}$, $m_{5}$, $m_{9}$, $t_{10}$	1699 (out of 3871) (43.8%)	196 (out of 434) (45.1%)

**Table 8:** Knockout impact of the selected transitions on the atherosclerosis process ($t_{109}$). In parenthesis—a change in firing probability is given as a percentage points difference to a reference firing value for $t_{109}$ presented in the first row (when nothing has been knocked-out).

Disabled Transitions/MCT Sets	$t_{109}$ Average Chance of Firing
Nothing Is Knocked Out in Net	27.29% (reference value)
$m_{11}$, $t_{28}$, $t_{43}$, $t_{120}$	2.71% (−24.58%)
$m_{11}$, $t_{28}$, $t_{43}$, $t_{116}$, $t_{120}$	2.72% (−24.57%)
$t_{120}$	2.92% (−24.38%)
$t_{116}$	4.05% (−23.24%)
$m_{11}$, $t_{28}$, $t_{43}$, $t_{116}$	5.10% (−22.19%)
$t_{25}$	26.82% (−0.47%)
$t_{12}$	27.1% (−0.19%)
$t_{73}$, $t_{113}$	27.16% (−0.13%)
$m_{11}$, $t_{28}$, $t_{43}$, $t_{73}$, $t_{113}$	27.16% (−0.13%)
$t_{113}$	27.20% (−0.09%)
$m_{1}$	27.21% (−0.08%)
$t_{51}$	27.24% (−0.05%)
$t_{116}$, $t_{120}$	0.0% (−27.29%)

## Data Availability

Not applicable.

## Supplementary Materials

The following are available at https://www.mdpi.com/article/10.3390/biology11030430/s1.
